# Secular trends in smoking in relation to prevalent and incident smoking-related disease: A prospective population-based study

**DOI:** 10.18332/tid/112459

**Published:** 2019-10-07

**Authors:** Philip Tonnesen, Jacob L. Marott, Børge Nordestgaard, Stig Egil Bojesen, Peter Lange

**Affiliations:** 1 Department of Sleep Medicine, Glostrup University Hospital, Copenhagen, Denmark; 2 The Copenhagen General Population Study, Herlev and Gentofte Hospital, Copenhagen University Hospital, Herlev, Denmark

**Keywords:** asthma, chronic obstructive pulmonary disease (COPD), prevalence of tobacco smoking, special populations, incidence of smoking

## Abstract

**INTRODUCTION:**

We examined changes in smoking habits in the general population according to prevalence and incidence of chronic diseases affected by smoking.

**METHODS:**

We included 12283 individuals enrolled from 2003 in the Copenhagen General Population Study and re-examined from 2014. Participants were classified as either healthy or suffering from chronic obstructive pulmonary disease (COPD), asthma, diabetes mellitus, heart disease or stroke.

**RESULTS:**

At entry, smoking prevalence was 15.4% in healthy participants, 29.8% with COPD, 15.8% with asthma, 21.7 % with diabetes mellitus, 17.2 % with ischemic heart disease/heart failure and 18.6% in participants with previous stroke. Smoking prevalence declined during the 10 years of observation. Among healthy subjects who developed one of the above mentioned diseases during follow-up, those who developed COPD had the highest initial smoking prevalence (51.5%). Quit rates were highest in those who developed asthma resulting in smoking prevalence of 8.2% versus 27.7% in COPD. After adjustment for age, smoking severity and genotype previously associated with heavy smoking (CHRNA3 rs1051730 AA), significant predictors of quitting were new diagnosis of ischemic heart disease/heart failure (OR=2.33, 95 % CI: 1.61–3.42), new diagnosis of asthma (OR=1.84, 95% CI: 1.18–2.90) and low number of pack-years.

**CONCLUSIONS:**

Individuals with prevalent smoking related diseases continued to smoke more than healthy individuals. Incident heart disease and asthma, but not incident COPD, stroke or diabetes were associated with a higher chance of quitting. Special focus on smokers with COPD, asthma, diabetes, stroke and ischemic heart disease/heart failure is warranted to decrease smoking prevalence in these groups. Smokers with a new diagnosis of diabetes, stroke and COPD need special smoking cessation support.

## INTRODUCTION

Tobacco smokers lose approximately 10 years of expected life duration compared to never smokers^1^. Long-term smoking is one of the most important preventable risk factors for morbidity and mortality, especially regarding chronic obstructive pulmonary disease (COPD), lung cancer and atherosclerotic cardiovascular disorders. Smokers with asthma have more symptoms and need a higher dose of inhaled steroids and more often experience faster disease progression^2^. COPD patients who quit smoking have a lower decline in lung function, lower morbidity and lower mortality compared with COPD patients who continue to smoke^3^. Also in diabetes, continuous smoking is a predictor of increased mortality^4,5^. Although smoking cessation is one of the most cost-effective interventions in medicine, it is still underused^6,7^.

Smoking prevalence in patients with asthma but not with COPD has almost halved in Sweden from 2005 to 2012^8^. In another study of 4636 smokers from 7 centers in North Europe followed for 10 years, 39 % stopped smoking. In this cohort asthma, wheeze, hay fever, chronic bronchitis, diabetes and hypertension did not significantly predict smoking cessation, but smokers hospitalized for ischemic heart disease (IHD) during the study period were more prone to stop smoking^9^.

As both presence and in particular incidence of smoking related disease may open a window of opportunity for motivation to quit, we found it of interest to examine secular trends in smoking prevalence over a period of 10 years in the general population. We compared smoking habits in smokers without chronic disease with individuals with either prevalent or incident COPD, asthma, diabetes mellitus (DM), stroke and heart disease, and explored the presence of these conditions as potential predictors of quitting.

## METHODS

### Study population

We identified 12283 individuals who participated in the second examination of the Copenhagen General Population Study from 6 February 2014 to 9 March 2017, who had also participated in the first examination 10 years earlier. This was a Danish contemporary population-based cohort study, which was approved by Herlev and Gentofte Hospital and the regional ethics committee (H-KF-01-144/01) and conducted according to the Declaration of Helsinki^10,11^. All participants provided written informed consent.

In Denmark, all individuals are assigned a unique identification number, at birth or at immigration, and recorded in the national Danish Civil Registration System. Based on age and sex, individuals living in the Capital Region of Denmark were randomly selected and invited to participate from the national Danish Civil Registration System to reflect the adult Danish population (response rate was about 45%). All participants completed a comprehensive questionnaire and underwent a physical health examination. Questionnaires were reviewed at the day of attendance by a healthcare professional and the participant.

In the analyses, we included the genetic variant CHRNA3 rs1051730, which in previous studies has been shown to be associated with higher tobacco consumption^12-14^. The study staff performing the genotyping were not aware of participants characteristics including their smoking status. The ABI PRISM 7900HT Sequence Detection System (Applied Biosystems Inc.) was used to genotype CHRNA3 rs1051730 with TaqMan assays. Genotyping was verified by DNA sequencing, and call-rates were above 99.8% as we performed re-runs twice.

In this paper we included 10251 subjects who all attended the first and second examination. In the analyses of smoking cessation, we only included 2563 individuals who were smokers at the first examination round.

### Definitions of smoking habits

- *Smokers:* self-reported daily smokers of any form of tobacco.
- *Ex-smokers:* individuals reporting having stopped smoking before the first examination.
- *Never smokers:* self-reported never smokers.
- *Quitters:* current smokers at first examination and former smokers at second examination.
- *Continued smokers:* current smokers at both surveys.

There was no biochemical verification of nonsmoker status.

### Definitions of prevalent chronic diseases

#### Healthy smokers

These were chosen at the first examinations as the reference group and defined according to the following: 1) Spirometry without presence of airflow limitation, which was defined as forced expiratory volume in 1 second (FEV_1_)/forced vital capacity (FVC) <0.7 at entry or follow-up; 2) No hospital admissions for COPD or asthma during the follow-up period; 3) No self-reported COPD or asthma at entry or at follow-up; 4) No self-reported medicine for asthma/COPD at entry or follow-up; 5) No self-reported IHD, heart failure (HF), stroke or diabetes mellitus (DM) at study entry; 6) No hospital admission in the follow-up period for IHD/HF, DM or stroke; and 7) No self-reported use of insulin or other medicine for DM at study entry or at follow-up.

#### COPD

Defined at study entry as one or more of the following: 1) Hospital admission for COPD before study entry; 2) Self-reported COPD at follow-up with time of diagnosing longer than the interval between the two examinations; 3) Self-reported medicine for asthma/COPD at study entry; and 4) No self-reported asthma.

#### Asthma

Defined at study entry as one or more of the following: 1) Hospital admission for asthma before study entry; 2) Self-reported asthma at study entry; 3) Self-reported medicine for asthma/COPD at study entry; and 4) No criteria fulfilling the above-mentioned COPD definition.

#### IHD and/or HF

Defined at study entry as either hospital admission for IHD or HF before study entry or self-reported previous ‘heart attack’ at study entry.

#### Stroke

Defined at study entry as either hospital admission for stroke before study entry or self-reported previous stroke at study entry.

#### DM

Defined at study entry as either hospital admission for DM before study entry or self-reported diabetes at study entry.

### Definitions of incident chronic conditions

#### New diagnosis of COPD

Defined as one or more of the following: 1) Hospital admission for COPD between entry and follow-up; 2) Self-reported COPD during follow-up with time of diagnosing shorter than the interval between the two examinations; 3) Self-reported medicine for asthma/COPD during follow-up; 4) No self-reported asthma during follow-up; and 5) No COPD at study entry according to the COPD definition above.

#### New diagnosis of asthma

Defined as one or more of the following: 1) Hospital admission for asthma between study entry and follow-up; 2) Self-reported asthma during follow-up; 3) Self-reported medicine for asthma/COPD during follow-up; and 4) No asthma at study entry according to the above asthma definition.

#### New diagnosis of IHD/HF

Defined as hospital admission for IHD or HF between study entry and follow-up and no IHD or HF at study entry according to the above definition.

#### New diagnosis of stroke

Defined as hospital admission for stroke between study entry and follow-up and no stroke at study entry according to the above definition.

#### New diagnosis of DM

Defined as one or more of the following: 1) Hospital admission for DM between study entry and end of follow-up; 2) Self-reported DM during follow-up; 3) Self-reported use of insulin or other medicine for DM during follow-up; and 4) No DM at study entry according to the above DM definition.

The ICD (International Classification of Diseases) codes used for the different diseases were: COPD (ICD-8: 491-492; ICD-10: J41-J44), asthma (ICD-8: 493; ICD-10: J45-J46), IHD/HF (ICD-8: 410-414, 427.09-427.11; ICD-10: I20-I25, I50), DM (ICD-8: 249-250; ICD-10: E10-E11, E13-E14), and stroke (ICD-8: 430-438; ICD-10: I60-I68, G45)^15^.

### Statistical analysis

For demographics, Pearson’s χ^2^-test and analysis of variance were performed for categorical and continuous variables, respectively. The quit rate was calculated as the proportion of smokers at 1st examination who reported being former smokers at 2nd examination. Stepwise model selection in multivariable logistic regression analysis for smoking cessation according to the Akaike Information Criterion (AIC) was performed. Possible explanatory variables were demographic variables (sex, age, years in school, body mass index), smoking related variables (early smoking debut, heavy smoking, pack-years), and CHRNA3 rs1051730 genotype. The stepwise regression analysis was initially performed on the entire data set and the resulting variables were considered possible confounding variables and included in each of the disease-specific analyses (Table 3).

## RESULTS

**Figure 1:**
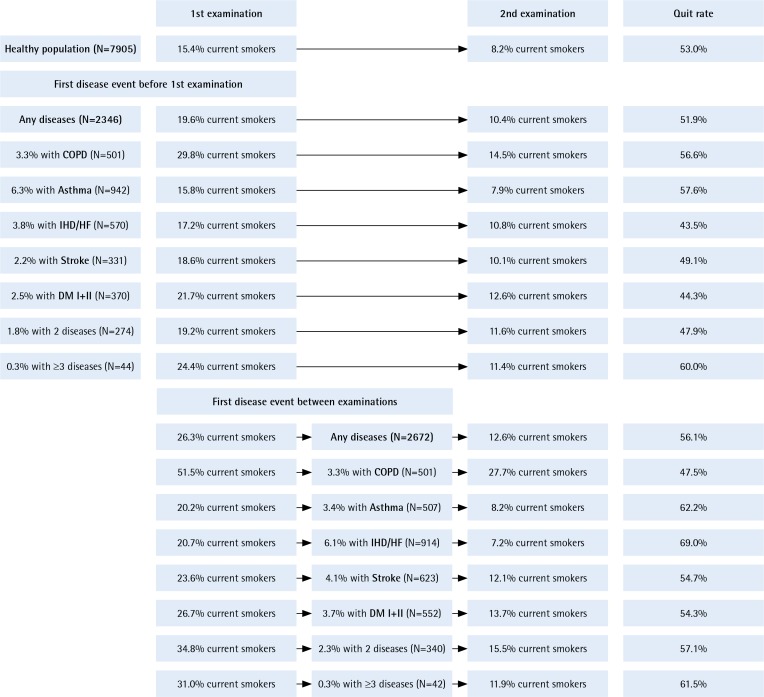
Smoking prevalence in healthy smokers and in the different disease groups at the 1st and the 2^nd^ examination for prevalent disease (upper part) and incident disease (lower part)

The total study population of individuals attending both the 1st and the 2nd examination of the Copenhagen General Population Study comprised 12283 individuals including 7905 healthy subjects and 2346 subjects with any of the investigated diseases already present at the first examination (Figure 1). Between the two examinations, 2672 experienced incidence of new disease. The upper part of Figure 1 shows smoking prevalence in the group of healthy individuals at both examinations. The middle part shows smoking prevalence in individuals with first disease event before 1st examination. The lower part shows smoking prevalence in subjects who were healthy at study entry but experienced a disease event between the two examinations (incident disease).

Among the 2346 participants with disease at study entry, smoking prevalence was higher than in the healthy participants with 15.8% among those with asthma and highest in those with COPD (29.8%), with in-between prevalences for DM (21.7%), IHD/HF (17.2%) and stroke (18.6%) (Figure 1). Smoking prevalence declined from the 1st to the 2nd study, in all subgroups. The 10-year quit rate was 53.0% for the healthy subjects compared with 51.9 % for subjects with any disease, and a quit rate of 57.6% in individuals with asthma and 56.6% in those with COPD (Figure 1).

Among the 2672 subjects who were healthy at study entry but experienced disease events between examinations, individuals with COPD had the highest initial smoking prevalence (51.5%) (Figure 1). In general, developing new disease was associated with a tendency towards higher quit rates especially among those who developed asthma (quit rate 62.2%). The lowest quit rate of 47.2% was observed in COPD.

Baseline demographics for the 2563 smokers at the 1st examination according to smoking status at follow-up 10 years later are shown in Table 1. Those who quit smoking had significantly lower pack-years and a higher debut age of smoking. They had a lower number of GP visits and more of them were characterized as healthy.

**Table 1:** Baseline demographics for the 2563 individuals who were smokers at the first examination in 2003–2014 according to smoking status at the final examination in 2014–2017

	*Smoking quitters*	*Continued smokers*	*p*
**General characteristics**	N=1300	N=1263	
Men, n/total (%)	576/1300 (44)	565/1263 (45)	0.86
Age (years), mean (SD)	54 (9)	53 (9)	0.51
Education, n/total (%)			0.23
<8	142/1296 (11)	148/1256 (12)	
8–10	708/1296 (55)	722/1256 (57)	
>10 years	379/1296 (29)	322/1256 (26)	
University	67/1296 (5)	64/1256 (5)	
BMI (kg/m^2^), mean (SD)	25 (4)	26 (4)	0.36
High physical activity in leisure time, n/total (%)	525/1292 (41)	501/1252 (40)	0.83
GP visits >6, n/total (%)	61/1013 (6)	104/991 (10)	<0.001
**Smoking related variables**			
Smoking debut before age 14, n/total (%)	104/1287 (8)	135/1257 (11)	0.026
Daily tobacco consumption (g/day), mean (SD)	14 (9)	17 (9)	<0.001
Years of smoking, mean (SD)	33 (10)	35 (10)	<0.001
Pack-years, mean (SD)	22 (18)	28 (19)	<0.001
CHRNA3 rs1051730 AA genotype, n/total (%)	133/1276 (10)	158/1239 (13)	0.08
**Health status**			
Healthy^a^	546/1300 (42)	484/1263 (38)	0.06
**First disease event before 1st examination**			
Any diseases	215/1300 (17)	199/1263 (16)	0.63
COPD	77/1300 (6)	59/1263 (5)	0.19
Asthma	76/1300 (6)	56/1263 (4)	0.13
IHD/HF	40/1300 (3)	52/1263 (4)	0.19
Stroke	27/1300 (2)	28/1263 (2)	0.91
DM I+II	31/1300 (2)	39/1263 (3)	0.33

Smoking quitters: current smokers at first examination and former smokers at second examination. Continued smokers: current smokers at both surveys.

a No COPD, asthma, IHD/HF, stroke or DM at any of the examinations.

Table 2 shows the characteristics of smokers at the first examination according to their health status. In general, those who either initially had or subsequently developed one of the chronic diseases were older, had a lower level of education, higher BMI (kg/m^2^), more pack-years and daily cigarettes smoked, and an earlier smoking start than healthy smokers.

**Table 2:** Characteristics in individuals with prevalent and incident chronic diseases for the 2563 individuals who were smokers at the first examination in 2003–2014

*Health status*	*Male sex*	*Age*	*School years*	*BMI kg/m^2^*	*Smoking debut before age 14*	*Tobacco/day ≥15 g*	*Pack-years*	*CHRNA3 rs1051730 AA genotype*
	*n/total (%)*	*mean (SD)*	*mean (SD)*	*mean (SD)*	*n/total (%)*	*n/total (%)*	*mean (SD)*	*n/total (%)*
Healthy^a^	438/1030 (43)	50 (9)	10.3 (1.8)	25 (4)	90/1023 (9)	523/1030 (51)	19 (16)	114/1014 (11)
**First disease event before 1st examination**								
Any diseases	189/414 (46)	57 (9)	9.6 (1.8)	27 (5)	49/410 (12)	266/414 (64)	30 (21)	38/402 (9)
COPD	58/136 (43)	58 (8)	9.3 (1.9)	26 (4)	15/136 (11)	106/136 (78)	37 (21)	14/134 (10)
Asthma	46/132 (35)	53 (10)	9.7 (1.9)	26 (5)	20/132 (15)	81/132 (61)	27 (19)	13/128 (10)
IHD/HF	64/92 (70)	59 (9)	9.3 (1.8)	27 (4)	15/90 (17)	49/92 (53)	30 (19)	11/88 (13)
Stroke	21/55 (38)	58 (10)	9.9 (1.6)	26 (4)	6/53 (11)	26/55 (47)	25 (19)	0/53 (0)
DM I+II	35/70 (50)	59 (8)	9.3 (1.7)	29 (6)	6/69 (9)	52/70 (74)	37 (24)	5/69 (7)
**First disease event between examinations**								
Any diseases	307/640 (48)	57 (9)	9.7 (1.8)	26 (5)	62/635 (10)	423/640 (66)	30 (20)	71/626 (11)
COPD	105/244 (43)	57 (9)	9.7 (1.8)	26 (5)	29/242 (12)	166/244 (68)	31 (20)	35/239 (15)
Asthma	34/98 (35)	55 (9)	9.7 (1.9)	26 (4)	9/98 (9)	69/98 (70)	31 (21)	11/97 (11)
IHD/HF	107/168 (64)	58 (9)	9.6 (1.8)	27 (4)	16/167 (10)	107/168 (64)	30 (20)	10/163 (6)
Stroke	66/128 (52)	58 (9)	9.7 (1.8)	25 (4)	10/127 (8)	76/128 (59)	26 (21)	12/124 (10)
DM I+II	72/140 (51)	56 (8)	9.6 (1.8)	30 (5)	10/138 (7)	100/140 (71)	33 (17)	17/138 (12)

a No COPD, asthma, IHD/HF, stroke or DM at any of the examinations.

Table 3 shows predictors for quitting smoking between the two examinations derived from a multivariable logistic step-wise regression analysis with focus on prevalent and incident diseases. All models included age, sex, pack-years, heavy smoking and CHRNA3 genotype. The most significant predictors of quitting were lower pack-years, new diagnosis of asthma (OR=1.84, 95% CI: 1.18–2.90, p<0.01) and new diagnosis of IHD/HF (OR=2.33, 95% CI: 1.61–3.42), p<0.001), whereas having one of these diagnoses at study entry did not influence quitting rate (Table 3). The level of education and physical activity in leisure time had no significant impact on quitting.

**Table 3:** Multivariable logistic regression analysis of predictors for quitting smoking between the two examinations for prevalent and incident diseases

	*OR ( 95% CI)*
**Confounding variables**	
Pack-years	
<10	1.00 (reference)
10–30	0.57 (0.44–0.75)***
≥30	0.44 (0.31–0.62)***
Heavy smoking (≥15 g tobacco/day)	0.81 (0.64–1.02)
Age (years)	1.01 (1.00–1.02)
CHRNA3 rs1051730 AA genotype	0.82 (0.64–1.05)
**Prevalent diseases**	
Multi-diseases	0.98 (0.56–1.71)
COPD	1.41 (0.95–2.10)
Asthma	1.30 (0.89–1.92)
IHD/HF	0.74 (0.46–1.18)
Stroke	0.86 (0.47–1.56)
DM I+II	0.80 (0.47–1.35)
**Incident diseases**	
Multi-diseases	1.52 (1.02–2.28)*
COPD	0.98 (0.72–1.34)
Asthma	1.84 (1.18–2.90)**
IHD/HF	2.33 (1.61–3.42)***
Stroke	1.28 (0.87–1.90)
DM I+II	1.35 (0.93–1.98)

OR: odds ratio. All models are adjusted for the confounding variable: pack-years, heavy smoking, age, and CHRNA3 rs1051730 AA genotype. Stepwise model selection based on Akaike Information Criterion was performed among the variables in the columns of Table 2.

* p<0.05;

** p<0.01;

*** p<0.001.

Changes in clinical characteristics between study entry and follow-up for the 1300 quitters and the 1263 continued smokers are shown in Table 1S in the Supplementary file. As expected, quitting smoking was associated with lower decline of FEV^1^ and weight gain.

## DISCUSSION

The main findings of the present study include the following. First, the smoking prevalence was higher among individuals with defined chronic diseases compared to healthy subjects. During the 10-year observation, smoking prevalence decreased by approximately 50% both in healthy subjects and in those with smoking related conditions. Most importantly, however, is our finding that incident diagnoses of IHD/HF and asthma were related to a higher chance of quitting compared with incident diagnosis of COPD, diabetes and stroke, despite weight increases in those who quit.

### Prevalent chronic disease and smoking

Presence of any of the investigated diseases at study entry was not associated with a higher chance of quitting compared to healthy smokers. Probably, many of the ever-smokers in the present study already quit smoking when they got the diagnosis and after that the quit rate was in the same range as in healthy smokers. Looking at the absolute smoking rates for individuals with prevalent asthma and IHD/HF in particular, they were at the same level as healthy subjects, whereas those with COPD had the highest smoking prevalence of 14.5%. In smoking cessation studies with COPD patients, the quit rates with all types of smoking cessation medication were lower compared to healthy smokers and especially the placebo quit rate is very low, indicating that COPD patients that smoke have more difficulties with stopping smoking^16^. It has also been reported that smoking cessation support is underused in smokers with COPD^6,7^. For example, among US patients with COPD, the average annual prescription rate of any smoking cessation medication from 2007 to 2012 was only 3.6%^6^. Thus, this seems to be an area where help towards smoking cessation should be much more offered.

In a Swedish study with a 7-year follow-up, smoking prevalence decreased from 11% to 6% (p<0.0001) in patients with asthma, but was almost unchanged in patients with COPD (28% to 26%, p=0.37)^8^. Compared with our results, the findings regarding asthma are similar, but the Swedish findings differ from ours regarding COPD, where we observed a quit rate of 56.6% with smoking prevalence decreasing from 29.8% to 14.5%. We cannot find any plausible explanation for this difference regarding COPD especially as Sweden and Denmark have a very similar healthcare system.

### Incident disease and smoking

The strongest predictor of quitting in our study was a new diagnosis of heart disease with OR=2.33. A new diagnosis of asthma was also significantly related to smoking cessation, whereas incident COPD, diabetes and stroke were not. It is not surprising that a new diagnosis results in smoking cessation and similar findings have been reported in the past. A Norwegian study with 1150 smokers followed from 2001–2 to 2007–8 reported that an incident diagnosis of asthma or COPD and IHD resulted in a quit rate of 50.6% for both diagnosis with an OR=1.7 for quitting^17^. Less effect on quitting was reported in a US population study with 11191 subjects without chronic disease at study entry who were followed from 1992 to 2006. An incident diagnosis of cancer and stroke had the highest impact on quitting smoking, with a smaller effect of IHD and COPD. Smoking rates for IHD decreased by 40% (from 24.5% to 14.9%) while for COPD smoking rates decreased by 19% (from 43.8% to 35.3%). This means that almost 60% of patients with IHD who smoke continued to smoke and supports the notion that an incident disease calls for a more aggressive approach by offering support and medication for smoking cessation^18^.

Patients with incident IHD in hospital based smoking cessation programs, as well as referral to cardiac rehabilitation, were strongly associated with increased smoking cessation rates after 6 months^19^. Applying this model for asthma, COPD, DM and stroke patients could be a way to increase quit rates.

### Other predictors of quitting

We found that smokers with higher pack-years had a markedly lower quit rates, which is in accordance with previous findings^9^. Although previous studies have suggested that the genotype CHRNA3 rs1051730 AA is associated with nicotine dependence^12-14^ and we observed that it was more prevalent among continued smokers than among quitters, this genotype was not significantly related to quitting, suggesting that its role is of minor importance compared with the role played by newly developed heart disease or asthma.

### Limitations

The present study has some limitations. As it is a prospective observational study with two examination sessions separated by 10 years, we do not have information on the exact time of quitting or why and how the participants quit smoking, i.e. if they used support and/or medication or were still using smokeless tobacco. In addition, we do not have information on their nicotine dependence or biochemical verification of quitting. However, as this was not a study with a focus on smoking but on several other health parameters, the participants had no strong reasons not to declare their real smoking status. An indirect verification that the quitters had reported their real smoking status is the observation of substantial weight gain in those who reported quitting compared with the continued smokers.

## CONCLUSIONS

Smoking rates declined substantially from 2003 to 2014 in our study population. However, individuals with prevalent smoking related diseases continued to smoke more than healthy individuals. Incident heart disease and asthma, but not COPD, stroke or diabetes, were related to a higher chance of quitting. Offering adequate smoking cessation treatment and focus on the window of opportunity in those with incident disease should be considered.

## CONFLICTS OF INTEREST

The authors have completed and submitted the ICMJE Form for Disclosure of Potential Conflicts of Interest and none was reported.
