# Supplementary material for: Secular trends in smoking in relation to prevalent and incident smoking-related disease: A prospective population-based study
Source: Tob Induc Dis. 2019 Oct 7;17:72. doi: 10.18332/tid/112459 (PMC6830353; doi:10.18332/tid/112459)
Supplement: Supplementary file 1 [file TID-17-72-s1.pdf]

Online Table 1. Changes in characteristics between first and second examination

	Smoking Quitters N=1300	Continued Smokers N=1263	P Value[1]	Smoking Relapsers N=142	P Value[2]
Annual decrease in FEV_1, mL - mean (SD)	31 (32)	37 (33)	<0.001	35 (34)	<0.001
Weight increase, kg - mean (SD)	4.9 (6.9)	0.5 (6.7)	<0.001	-1.8 (6.3)	<0.001
Weight increase of more than 10 kg – no. (%)	262/1296 (20)	85/1261 (7)	<0.001	3/142 (2)	<0.001
Weight decrease of more than 10 kg – no. (%)	20/1296 (2)	60/1261 (5)	<0.001	11/142 (8)	<0.001
Increase in BMI, kg/m^2 - mean (SD)	2.2 (2.4)	0.7 (2.3)	<0.001	-0.1 (2.2)	<0.001
Increase in systolic blood pressure, mmHg - mean (SD)	2 (20)	0 (20)	0.015	-2 (15)	0.015
Started taking blood pressure medication – no. (%)	282/1111 (25)	225/1065 (21)	0.022	20/114 (18)	0.023
Hypertension onset – no. (%)	245/648 (38)	206/639 (32)	0.042	16/75 (21)	0.006
New admissions for COPD – no. (%)	73/1265 (6)	78/1233 (6)	0.62	2/140 (1)	0.06
New admissions for Asthma – no. (%)	23/1276 (2)	17/1247 (1)	0.47	2/139 (1)	0.67
New admissions for IHD or heart failure – no. (%)	117/1262 (9)	53/1215 (4)	<0.001	6/136 (4)	<0.001
New admissions for stroke – no. (%)	73/1281 (6)	62/1244 (5)	0.48	9/141 (6)	0.63
New admissions for DM II – no. (%)	31/1284 (2)	44/1243 (4)	0.12	3/141 (2)	0.21
New admissions for DM I+II – no. (%)	33/1283 (3)	48/1242 (4)	0.08	4/141 (3)	0.18

Smoking quitters: Current smokers at first examination and former smokers at second examination. Continued smokers: Current smokers at both surveys. Smoking relapsers: Former smokers at first examination and current smokers at second examination. [1]Compares smoking quitters with continued smokers. [2]Compares smoking quitters, continued smokers and smoking relapsers.

© 2019 Tonnesen P.
